# A time-lagged effect of conspecific density on habitat selection by snowshoe hare

**DOI:** 10.1371/journal.pone.0190643

**Published:** 2018-01-10

**Authors:** Toshinori Kawaguchi, André Desrochers

**Affiliations:** Centre d’étude de la forêt, and Département des sciences du bois et de la forêt, Université Laval, Québec, Québec, Canada; Liverpool John Moores University, UNITED KINGDOM

## Abstract

Ideal free distribution theory predicts that increased conspecific density redistributes individuals to low-density, suboptimal habitat. However, possible lags in response to population density remain poorly documented. Snowshoe hares (*Lepus americanus*) may exhibit density-dependent habitat selection due to its marked variation in population density. Based on 11 years (2004–2014) of snow tracking in Quebec (Canada), we investigated snowshoe hares’ short-term and delayed habitat selection responses to population density. We predicted that at high densities, hare distribution expands into low-density habitat, thus weakening the association between hares and high-density habitat. We surveyed hare tracks along 95 km of transects on average each year and georeferenced 14,240 tracks. We used Generalized Estimating Equations for track count per 100 m transect segment as a function of the proportion of different forest age classes (0–20 y, 20–40 y and 40–80 y) within 50 m of the segments. We used model coefficients for each age class as a measure of habitat preference, and modeled those coefficients as a function of a population density index in current and previous winters. Coefficients for 20- to 40-y-old forests were positive each year, indicating that this habitat was preferred. The association between track counts and 20- to 40-y-old forest significantly declined with density during the previous winter, suggesting that hare spread from preferred forest with a lagged response to density. To our knowledge, no previous empirical studies have documented a lagged habitat selection response to population density. Time lags offer possible explanation for documented deviations from ideal free distribution models.

## Introduction

Habitat selection is a response to a large variety of stimuli, such as vegetation structure and composition, predation risk [1], weather [2], and conspecific population density [3]. The effect of conspecific population density on habitat selection has been documented for various taxa, including mammals (e.g., fat sand rat *Psammomys obesus* [4], domestic sheep *Ovis aries* [5]) and birds (e.g., brown-headed cowbird *Molothrus ater* [6]). The ideal free distribution model [3] is based on animals moving freely among habitats of different quality in order to maximize their fitness [7]. According to Morris’s isodar model [7], fitness decreases with increased density, which may lead individuals to move from high-density to low density habitat.

Time lags are often observed in processes such as species redistribution following landscape changes [8], local extinction after deforestation [9], species invasion [10], and population dynamics [11,12]. Time lags can be caused by: 1) long processing times after stimulus perception [9], 2) intervening processes between two processes of interest [13], and 3) feedbacks [14].

While several empirical studies have documented the ideal free distribution [15, 16], deviations from ideal free model have also been reported [17,18]. Such deviations have been interpreted as the result of limited perceptual constraints [19], despotic behavior [20] or site familiarity [21]. Ideal free distribution may also be delayed by events occurring between the increase in the density (stimulus) and the establishment of the ideal free distribution. Such events may include the perception of a decrease in fitness, or the discovery of available nearby habitats [22]. Even though the ideal free distribution is unlikely to be instantaneous, no one has, to our knowledge, examined the lag in responses to density shifts, which may account for apparent deviation from ideal free distribution.

We documented population dynamics of snowshoe hare (*Lepus americanus*) over the course of 11 winters, evaluating the effect of density on snowshoe hare habitat selection, and assessing delays in response to density changes. More specifically, based on winter track counts, we measured the strength of association between hares and forest stands of different age classes to identify the most and least preferred habitats. We chose snowshoe hares because of their strong population fluctuations [23, 24] and reported deviations from an ideal free distribution [17]. We predicted that at high population densities, the spatial distribution of snowshoe hares expands into less preferred habitat, thus weakening the association between hares and the preferred habitat. We expect lags in snowshoe hare responses because of likely intervening processes such as the discovery of nearby habitat satisfying the understory cover requirement [25]. Therefore, we predicted that the distribution shift lag would be stronger in the winter following the high density trigger rather than in the current winter. In addition to population density, habitat selection by herbivores is greatly influenced by predation risk, resulting in varying responses for a particular habitat type [26]. Lynx (*Lynx canadensis*), red fox (*Vulpes vulpes*) and American marten (*Martes americana*) prey on snowshoe hare [23,24,27,28]. We hypothesized that predation risk modulates habitat selection by hares, in addition to density-dependent processes.

## Methods

Snow tracking surveys were carried out at the Montmorency Forest, a 66 km^2^ boreal forest approximately 80 km North of Quebec City (47°20’N, 71°10’W), Canada. A combination of clear-cuts and selective cuts is applied over the majority of the study area. Forest stand composition is 55% mature (more than 40-y-old), 25% in regeneration (21- to 40-y-old) and 20% young forest (less than 20-y-old). Stand location shifted with time due to timber harvest and forest stand succession: mean stand age remained stable throughout the study period (43.3 ± 2.0 y; mean ± sd, range 0–114 y). A dense road network is present, with about 150 km of roads, i.e., more than 2 km/km^2^. In winter, several forestry roads are groomed by machinery for cross-country ski trails. Elevation ranges from 650 m to 1000 m. Between 1999 and 2011, the annual mean temperature was 0.3°C and total annual precipitation was 1417 mm (33% as snow). During the same year interval, maximum snow depth at the study site’s weather station ranged from 62 cm to 146 cm [29].

Balsam fir (*Abies balsamea*) dominates second-growth mature forest stands. Black spruce (*Picea mariana*), white or paper birch (*Betula papyrifera*), trembling aspen (*Populus tremuloides*), and white spruce (*P*. *glauca*) are also common. Recent (less than 5-y-old) clear-cuts are generally colonized by red raspberry (*Rubus idaeus*), balsam fir, and white birch [30].

We conducted snow tracking each winter (20 Dec—31 Mar) from 2004 to 2014. We counted tracks along a network subset of road network that included about 150 km of roads, 40 km of trails, and 60 km of straight line transects inside forest stands (refer to Kawaguchi et al. 2015 for the spatial distribution of the transect lines [31]). The roads surveyed were not snow-plowed. Transect length varied with weather, time of day, and personnel availability (Table 1). Off-trail transects were randomly selected from a systematic grid covering the entire study area at the beginning of each winter. Selected transects that had been surveyed in the previous year were removed during the selection process. Selected transects were surveyed only once per year to cover the largest area possible. The tracks were surveyed along transects of 94.73 km ± 30.57 km (mean ± sd) (Table 1). We surveyed transects within a 24 to 72 hours after each snowfall of more 3 cm. However, the sampling was not performed in absence of strong wind (> 20 m/s) after the snowfall. All tracks of hare, marten, lynx and fox that were within a visually estimated 2 m range of the transect lines were recorded into a GPS receiver. Conspecific tracks that were within 3 m of a recorded track were ignored. The snow tracking study at the Montmorency Forest was approved by the Université Laval, which holds a long-term lease of the entire study area for educational purposes.

**Table 1:** Sampling effort for snow-tracking of snowshoe hare (*Lepus americanus*), fox (*Vulpes vulpes*), lynx (*Lynx canadensis*) and marten (*Martes americana*) in the Montmorency Forest, southern Quebec (Canada), 2004–2014.

Year	km sampled		Hare		Predator			
	On road/trails	Off-trail	100-m segment (*n*)	Track count	400-m segment (*n*)	Fox	Lynx	Marten
2004	34	19	527	597	121	10	38	65
2005	61	6	671	390	159	18	17	59
2006	52	23	751	425	183	50	52	85
2007	72	17	890	943	209	50	55	136
2008	99	24	1234	1684	289	64	89	314
2009	59	12	715	344	166	114	0	93
2010	93	12	1055	834	252	121	1	98
2011	112	14	1263	1937	295	155	0	149
2012	93	42	1352	2896	312	188	31	183
2013	113	20	1325	3147	320	113	14	100
2014	52	13	653	1043	155	71	14	25
Total	840	202	10436	14240	2461	954	311	1307
Mean	76	18	949	1295	224	87	28	119
sd	27	9	306	996	72	56	28	78

sd: standard deviation.

We processed snowshoe hare track and transect data with ArcGIS (Version 10.1, ESRI 2012) and split the transects into 100-m segments, totaling 10,436 100-m segments for the entire study (Table 1). We counted tracks along each transect segment, and generated buffers with a radius of 50 m. Because the winter home range of snowshoe hare averages 2 ha in the study region [32], we considered 2 ha of resulting buffer size as a meaningful sampling unit size. Within each buffer, we calculated the mean age of forest stands, slope (the difference between maximum and minimum elevation), mean elevation, and the proportions of the area occupied by 3 habitat types, based on forest stand age (young: 0- to 20-y-old, regenerating: 20- to 40-y-old, mature: 40- to 80-y-old). Older forest stands (older than 80-y-old) were rare and not included in the analyses. Since buffers occasionally included roads, rivers and lakes, we also calculated the percentage of vegetated area inside each buffer. For predator tracks, we georeferenced the tracks to its corresponding transect and split transects into 400 m segments, totaling 2,461 transects segment. We assessed the presence of every predator track found in these segments.

To obtain indices of hare population density, we modelled track counts using the year as a categorical effect based on Generalized Estimating Equations (GEEs) with a negative binomial distribution and a log link function by using the geeM package (Version 0.7.4) in the R software [33]. The dispersion parameter was estimated by using the MASS package [34]. The year-effect obtained in this way is highly correlated with annual pelt sales from the same region in other species including weasels [30]. A negative binomial distribution was preferred over Poisson distribution due to large numbers of zero counts in our data. GEEs allowed us to account for spatial autocorrelation within transects [35]. The population index model included the following covariates: hours of exposure since the last snow fall, mean stand age, squared stand age, variance of stand age, slope, mean elevation, mean temperature in the previous 24 hours, transect type (road vs off-road), proportion of vegetated area. Month and year were considered as a categorical variable. Squared stand age was added because hare habitat use pattern showed a peak for 40 y stand age [36]. We integrated transect type (trail vs off-trail) into each model, because of the possible responses of snowshoe hare to roadside vegetation and openness [25,37]. The month variable was also included into the model to account for potential of hare population declines over the winter [38]. To account for the predation risk in the study area, the proportion of 400-m transect segments occupied by any type of predator tracks was calculated for each year.

To measure the strength of the hare-habitat association, we used a negative binomial track count model with GEEs. Explanatory variables were the same as for the population index model, except for the proportions of the area of three types of forest stand age instead of mean stand age and the squared age. We used stand age classes to avoid mixing preferred (regenerating stands, [36,39]), and non-preferred habitats. Relationships between the proportion of each habitat type and indexed vegetation density are presented in S1 Appendix).

A habitat use model was developed for each year, and model estimates for the proportion of each habitat was used as measures of habitat preference. To compare immediate and delayed effects of snowshoe hare population density on habitat preference, we used linear models for the preference as a function of population density in current and previous winters separately and combined as well as predation risk. To account for the accuracy heterogeneity of the estimates, the measured preference indices were weighted. Weights were calculated as *w*_*i*_ = (1/SE_i_)/(1/SE_1_ + 1/SE_2_ + … + 1/SE_k_) where *w*_*i*_ is a weight for measured habitat preference i at a given year, and SE is a standard error of estimated coefficient of habitat variable. Since we used lagged effects, we excluded the habitat preference in 2004 (initial year) from the analysis. The model with the highest adjusted *R*^*2*^ was considered as the best model. All statistical analyses were conducted in the R statistical environment [40] (Version 3.2.1).

## Results

**Fig 1:**
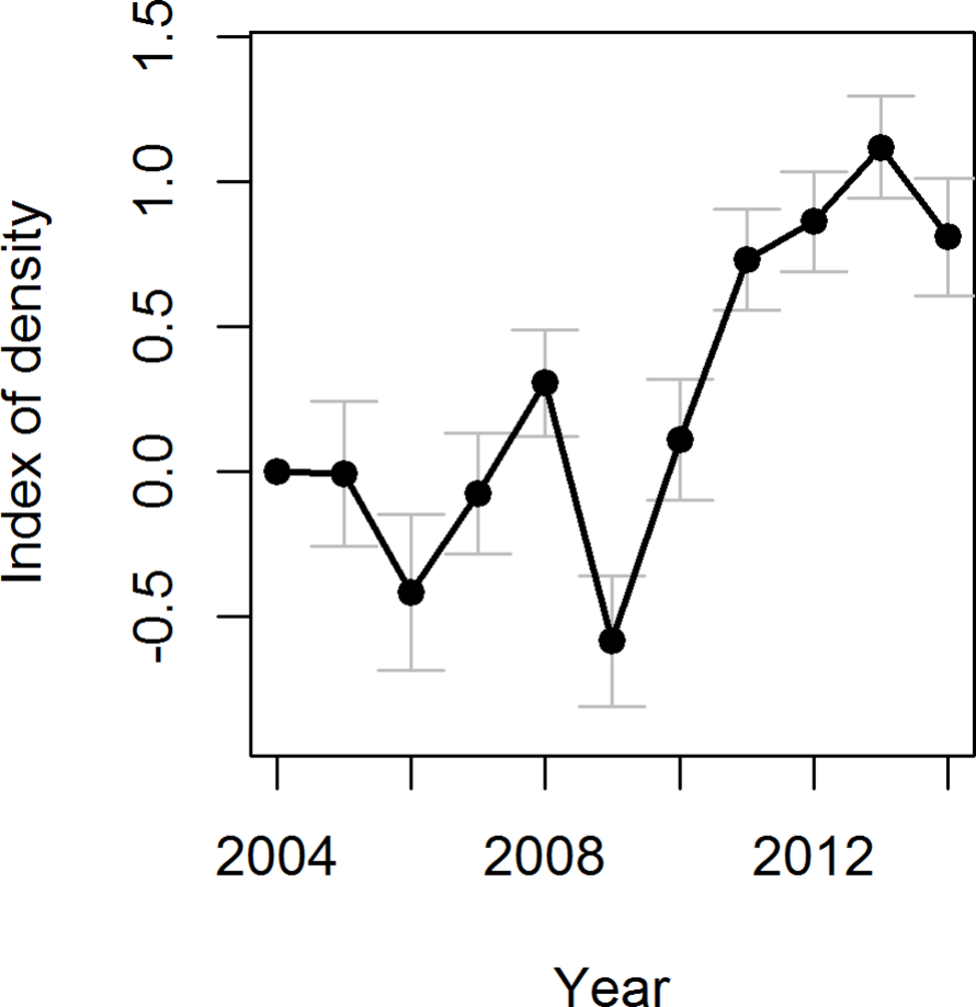
Estimated population index of snowshoe hare over 11 years from 2004 to 2014. The index was developed from year effect coefficients estimated from Generalized Estimating Equations (GEE). Vertical bars indicate standard errors.

A total of 14,240 snowshoe hare tracks in total were recorded (annual range: [344, 3,147]). Annual track counts per kilometer were 12.4 ± 6.3 (mean ± sd) and varied from 4.8 (2009) to 23.7 (2013) (Fig 1; S1 Table). The correlation between population indices of a given year and its previous year was high (*r =* 0.6), but not significant (*n* = 10, *P* = 0.06), indicating moderate positive correlation between the two indices. Annual track counts per kilometer for martens, lynx and foxes were 1.2 ± 0.5 (mean ± sd) [range: 0.4, 2.6], 0.3 ± 0.3 (mean ± sd) [range: 0.0, 0.72] and 0.9 ± 0.5 (mean ± sd) [range: 0.2, 1.6] respectively. The proportion of transect segments with predator tracks ranged from 0.296 to 0.574.

The association between track counts and the proportion of 0- to 20-y-old forest stands was negative for each year (range of model estimates: [-0.011, -0.001]; S2 Table), suggesting that this habitat was the least preferred. In contrast, the relationship between track counts and the proportion of 20- to 40-y-old stands was positive each year (range: [0.001, 0.012]; S2 Table), suggesting that 20- to 40-y-old stands were most preferred. Relationships between track counts and the proportion of 40- to 80-y-old stands were either positive or negative (range: [-0.005, 0.004]; S2 Table), depending on the year.

**Fig 2:**
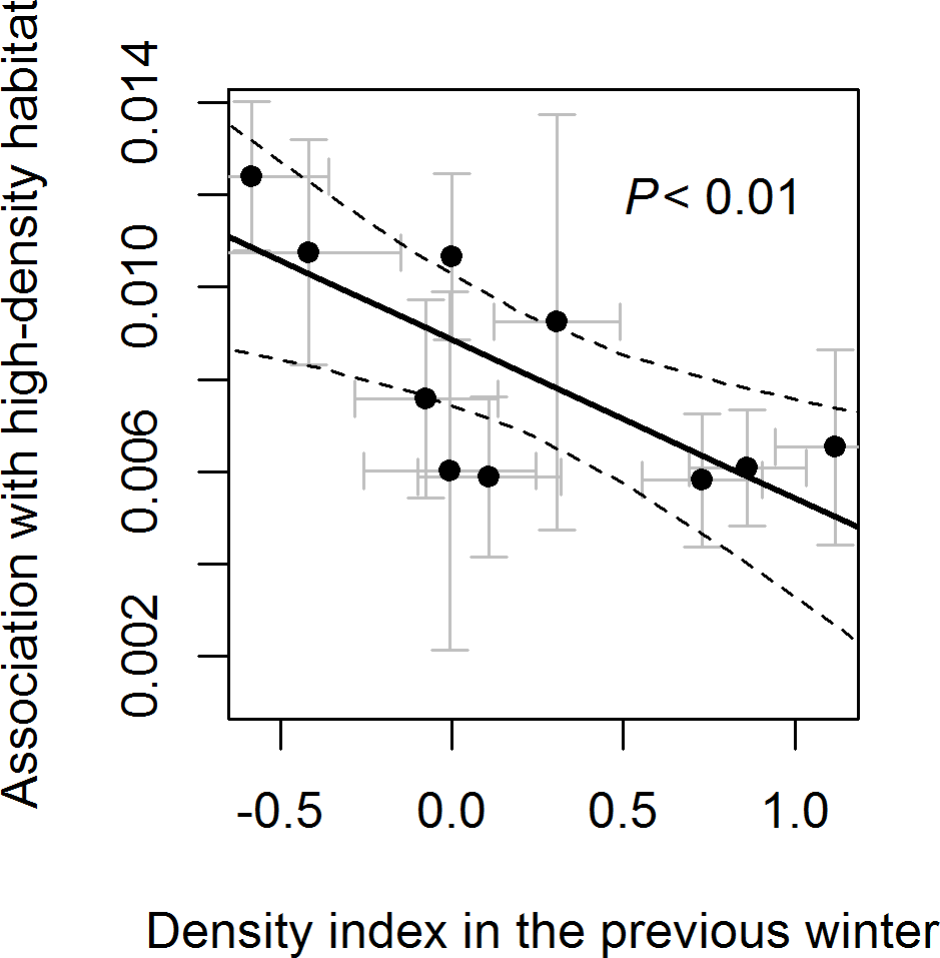
Association of snowshoe hares with the high-density habitat explained by time-lag effects of the density index. High-density habitat indicates the 20- to 40-y-old habitat. Points with standard error bars indicate model coefficients. Dashed lines indicate 95% confidence bands of the fitted regression line values.

The lag model for the response to 20- to 40-y-old forest stands performed best among the candidate models. The lagged effect of density was significantly negative, suggesting that hare less frequently used 20- to 40-y-old forest in response to higher density in the previous winter (Table 2; Fig 2). In the current model, the immediate effect of density was also significantly negative. The predator model had the worst performance among the candidate models and it was not found to be significant (Table 2). In other habitat types (0- to 20-y-old forest and 40- to 80-y-old forest), immediate, lagged and predator effects were not significant, but the predator and lagged models exhibited the highest adjusted R^2^ in 0- to 20-y-old forest and 0- to 80-y-old forest respectively (Table 2; Fig 2).

**Table 2:** Estimated effects of current and lag density (previous winter) and predation risk on habitat selection of snowshoe hares in the Montmorency Forest, Quebec, 2004–2014 (*n* = 10).

Model	Adjusted *R*^*2*^	Model estimates (β ± s.e)			
		Intercept	Current density	Lag density	Predator
*a) Models for 0-20y habitat preference*					
Predator	0.15	-0.0141±0.0012*	-	-	0.0002±0.0019^NS^
Lag	-0.09	-0.0061±0.0012***	-	0.0009±0.0019^NS^	-
Current	-0.1	-0.0062±0.0012***	0.0008±0.0019^NS^	-	-
Current +Lag	-0.25	-0.0061±0.0012***	0.0004±0.0019^NS^	0.0007±0.0011^NS^	-
*b) Models for 20-40y habitat preference*					
Lag	0.55	0.0089±0.0012***	**-**	-0.0034±0.0019***	**-**
Current + Lag	0.51	0.0092±0.0012***	-0.0011±0.0019^NS^	-0.0027±0.0011^NS^	-
Current	0.38	0.0094±0.0012***	-0.0032±0.0019*	-	-
Predator	-0.07	0.0109±0.0012*	-	-	-0.0001±0.0019^NS^
*c) Models for 40-60y habitat preference*					
Lag	-0.1	-0.0012±0.0012^NS^	-	0.0006±0.0019^NS^	**-**
Predator	-0.11	0.0006±0.0012^NS^	-	-	0±0.0019^NS^
Current	-0.12	-0.001±0.0012^NS^	0±0.0019^NS^	-	-
Current + Lag	-0.23	-0.0009±0.0012^NS^	-0.0009±0.0019^NS^	0.0011±0.0011^NS^	-

a) The effects on 0–20 y habitat preference of hare. b) The effects on 20–40 y habitat preference. c) The effects on 40–60 y habitat preference. Positive estimates indicate a greater association at higher density. Significant model estimates are shown in bold. Adjusted *R*^2^ values can be negative, because unlike raw *R*^2^, they are penalized by the number of parameters.

*** Indicates that the p value of the model estimate was below 0.01

* Indicates that the p value of the model estimate was less than 0.05 and more than 0.01.

NS indicates that the p-value of the model estimate was more than 0.05

## Discussion

Snowshoe hares wintering at the Montmorency Forest responded spatially to their population density with a lag of one year. The models including delayed effects explained the dynamic associations of hares with preferred habitats better than the model based on the immediate responses to population density. In contrast, population density poorly explained variation in hare association to forest stands that were 40- to 80-y-old and 0- to 20-y-old. Habitat use by snowshoe hare is known to vary seasonally [41] and thus these findings may be applicable to the winter.

The preference for forest stands that were 20- to 40-y-old was consistent with other studies [36, 39]. The avoidance of hares of 0- to 20-y-old forest stands was also consistent with past studies [39, 42]. This could be explained by the fact that saplings in those stands were mostly covered by snow, thus offering few opportunities for foraging and increased high predation risks.

We interpret the signs of the model estimates in 20- to 40-y-old forest stand associations as evidence for an overflow of individuals from the preferred habitat, in response to changes in population density. The lagged response of hares can be interpreted as a ‘buffer effect’ [43,44]. The apparent overflow of hares from high-density habitat was possibly delayed by a lag in the perception of stimuli (increased density) or by the discovery of nearby available habitat.

The immediate response to density by snowshoe hares was also significantly negative. Thus, the response to density by snowshoe hares was not entirely lagged, and this may reflect spatial variation in the availability of nearby alternative habitat.

Positive lagged effect coefficients of the 0- to 20-y-old and 40- to 60-y-old forest stands were expected to explain a lag in the shift of snowshoe hare use from the preferred habitat to the least preferred habitat. Contrary to our expectations, lagged effects of density in those habitats were not significant, suggesting that hares dispersing from their preferred habitat did not readily move toward into those habitats. This pattern could be attributed to higher mortality in low-density habitat, potentially offsetting the detection of increased habitat use by dispersing hare. As younger forest stands were more open and often partly covered by a thick layer of snow [45], hares in this habitat would be more vulnerable to predators such as Canada lynx. Immediate and deferred costs of dispersal are known to lower survival rates [46].

We found no relationship between habitat preference and predation risk index, as inferred from the presence of predator tracks. This result suggests that predation risk did not significantly influence hare habitat selection at the stand scale, and is consistent with other findings [47].

Deviations from the ideal free distribution have been observed in numerous empirical studies on density-dependent habitat selection. For example, a study in northwestern Ontario based on the isodar model indicated that snowshoe hares exhibited subtle density-dependent habitat selection, with a large residual variation [17]. In an Idaho study, local hare colonization was not enhanced by greater densities in neighboring areas [18]. As demonstrated here, integrating a time-lag effect can improve the explanatory power of density-dependent habitat selection models, possibly explaining the observed deviations from ideal free distribution models.

## Supporting information

S1 Appendix Relationships between proportion of each of the three habitat types and vegetation density indices. (DOCX)

S1 Table Model coefficients of the model for indexing population density of snowshoe hare. (DOCX)

S2 Table Model coefficients for effects of proportion of each habitat type. (DOCX)

S1 Data Track counts data of snowshoe hare in the southern Quebec. 2004–2014.
(XLSX)

S2 Data Track presence data of predator species in the southern Quebec. 2004–2014.
(XLSX)
