# Supplementary material for: A time-lagged effect of conspecific density on habitat selection by snowshoe hare
Source: PLoS One. 2018 Jan 10;13(1):e0190643. doi: 10.1371/journal.pone.0190643 (PMC5761860; doi:10.1371/journal.pone.0190643)

S1 Appendix: Relationship between vegetation densities and the three selected habitat types.

**Abstract:** In this appendix, we present the relationship between each of the three habitat types and the two types of vegetation density index: a) the density of low vegetation (0-1.5 m above ground) and b) vegetation density high above the ground (2-4 m above ground). The objective of this appendix is to demonstrate how vegetation density, an indicator of habitat quality, differs among the three types of habitats.

**Method:** Details regarding the vegetation density measurement method were presented in our previous study (Kawaguchi and Desrochers, in press). A brief description is provided below.

In 2012, we estimated the density of low vegetation (0-1.5 m above ground) along the same line transects used for snow tracking, but during a period without snow (August to November). The density of low vegetation was visually estimated as the proportion of ground covered by live herbaceous shrub and conifer vegetation below 1.5 m above ground. All vegetation located within a visually estimated 2 m range on each side of the line transect was assessed, along 50 m intervals. The resulting understory cover plots were 4 m x 50 m rectangles.

The vegetation density index was obtained high above the ground with an Airborne Light Detection and Ranging (LiDAR) image. We used a LIDAR reduction rate with a first return penetration rate (PN) between 2 m and 4 m above ground as an index of vegetation density above deep snow.

The Pearson product-moment correlations were calculated for every possible combination of the two vegetation densities and the three habitat types.

**Results**: The 0-20 y habitat was positively associated with the density of low vegetation (Figure S5-1); the 20-40 y habitat was positively associated with the density of high vegetation; and the 40-60 y habitat was negatively associated with both densities.

**Discussion:** The 0-20 y habitat had more vegetation cover on the ground and the 20-40y habitat had more vegetation cover 2 m above the ground or higher. Snow depth at some of the sampling locations in our study site was greater than 150 cm. The vegetation cover on the ground was therefore not available for snowshoe hares, as it was buried by deep snow.(Kawaguchi and Desrochers, in press).

**Reference**:

Kawaguchi, T. and A. Desrochers. in press. Short-term changes of spatial distribution pattern of an herbivore in response to accumulating snow. Canadian journal of the Zoology.

**Figure S5-1**. Relationships between habitat types and vegetation density: a) Proportion of 0-20 y habitat vs low vegetation index; b) Proportion of 0-20 y habitat vs high vegetation index; c) Proportion of 20-40 y habitat vs low vegetation index; d) Proportion of 20-40 y habitat vs high vegetation index; e) Proportion of 40-60 y habitat vs low vegetation index; f) Proportion of 40-60 y habitat vs high vegetation index.


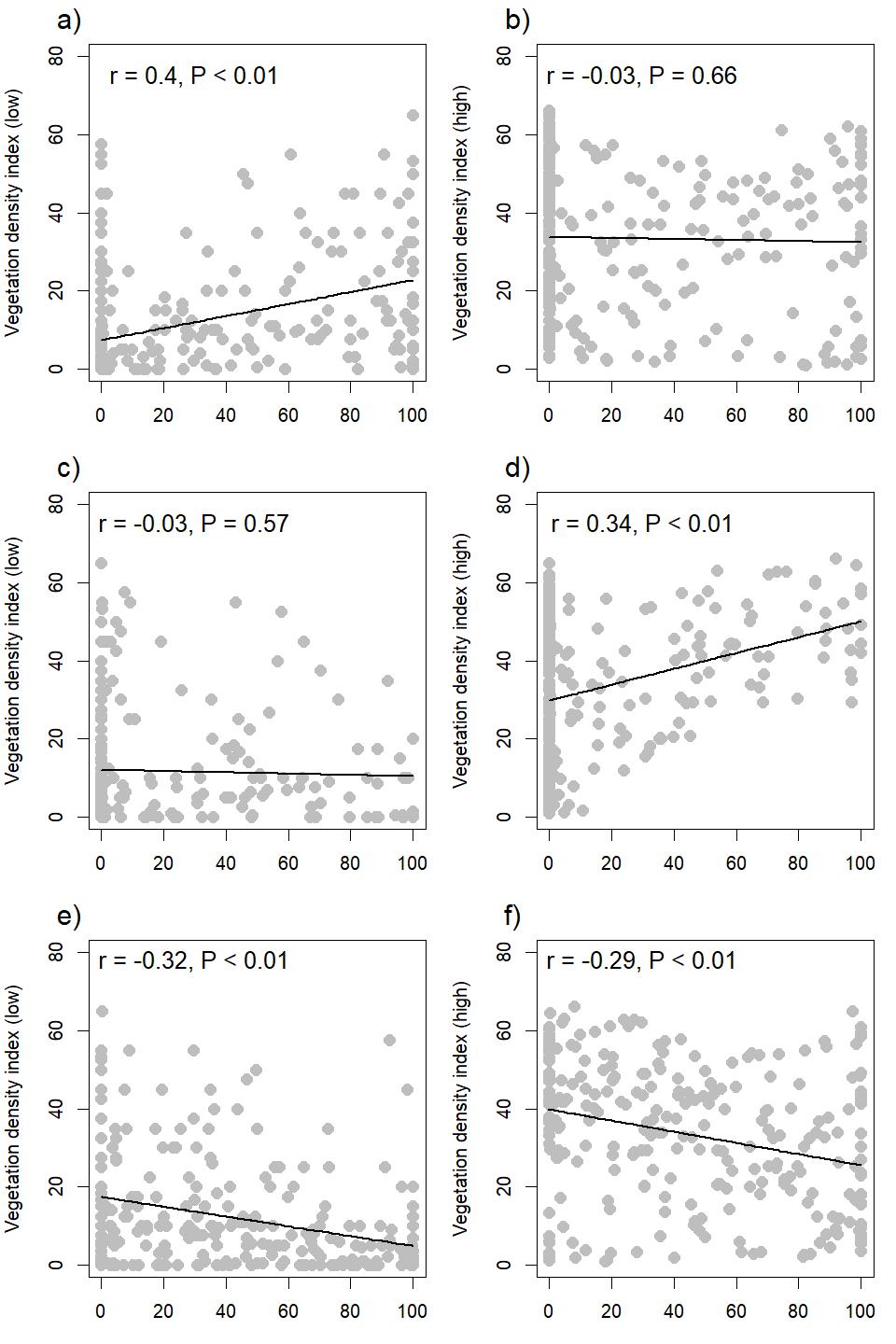

Supplement: S1 Appendix — (DOCX) [file pone.0190643.s001.docx]
