# Supplementary material for: A time-lagged effect of conspecific density on habitat selection by snowshoe hare
Source: PLoS One. 2018 Jan 10;13(1):e0190643. doi: 10.1371/journal.pone.0190643 (PMC5761860; doi:10.1371/journal.pone.0190643)
Supplement: S1 Table — (DOCX) [file pone.0190643.s002.docx]

**S1 table. Model coefficients of the model for indexing population density of snowshoe hare.** Estimation of year effect of each year used 2004 as the reference year. Estimation of month effect used January as the reference month.

| Variable | Estimate | Standard error | P value |
| --- | --- | --- | --- |
| Intercept | -1.543 | 0.4304 | <0.01 |
| Exposure time | -0.004 | 0.0027 | 0.13 |
| Year effect 2005 | -0.007 | 0.251 | 0.98 |
| Year effect 2006 | -0.417 | 0.2685 | 0.12 |
| Year effect 2007 | -0.075 | 0.2091 | 0.72 |
| Year effect 2008 | 0.305 | 0.1836 | 0.1 |
| Year effect 2009 | -0.584 | 0.2248 | <0.01 |
| Year effect 2010 | 0.109 | 0.208 | 0.6 |
| Year effect 2011 | 0.729 | 0.1742 | <0.01 |
| Year effect 2012 | 0.862 | 0.1715 | <0.01 |
| Year effect 2013 | 1.118 | 0.1765 | <0.01 |
| Year effect 2014 | 0.809 | 0.2008 | <0.01 |
| Mean age | 0.053 | 0.0057 | <0.01 |
| Mean age^2^ | -0.001 | 1.00E-04 | <0.01 |
| Mean temperature | 0.005 | 0.0074 | 0.51 |
| Slope | -0.015 | 0.0027 | <0.01 |
| Average altitude | 0.001 | 4.00E-04 | 0.12 |
| Variance of age | -0.001 | 1.00E-04 | <0.01 |
| Vegetated area | 0.01 | 0.0018 | <0.01 |
| Transect type | 0.597 | 0.0832 | <0.01 |
| Month effect Feb | -0.434 | 0.1067 | <0.01 |
| Month effect Mar | -0.639 | 0.1254 | <0.01 |
| Month effect Dec | 0.96 | 0.2874 | <0.01 |
