# Supplementary material for: A time-lagged effect of conspecific density on habitat selection by snowshoe hare
Source: PLoS One. 2018 Jan 10;13(1):e0190643. doi: 10.1371/journal.pone.0190643 (PMC5761860; doi:10.1371/journal.pone.0190643)
Supplement: S2 Table — (DOCX) [file pone.0190643.s003.docx]

**S2 table**. Model coefficients for effects of proportion of each habitat type. Estimates were shown for three habitat types: a) 0- to 20-y, b) 20- to 40-y and c) 40- to 60-y.

| YEAR | Estimates | Standard error | *P* value |
| --- | --- | --- | --- |
| 1. *0- to 20-y* | | | |
| 2004 | -0.01 | 0.003 | <0.01 |
| 2005 | -0.011 | 0.003 | <0.01 |
| 2006 | -0.002 | 0.003 | 0.48 |
| 2007 | -0.008 | 0.003 | <0.01 |
| 2008 | -0.002 | 0.002 | 0.4 |
| 2009 | -0.007 | 0.004 | 0.04 |
| 2010 | -0.008 | 0.002 | <0.01 |
| 2011 | -0.008 | 0.002 | <0.01 |
| 2012 | -0.004 | 0.002 | 0.02 |
| 2013 | -0.004 | 0.002 | 0.03 |
| 2014 | -0.009 | 0.004 | 0.02 |
|  |  |  |  |
| 1. *20- to 40-y* | | | |
| 2004 | 0.001 | 0.006 | 0.86 |
| 2005 | 0.011 | 0.002 | <0.01 |
| 2006 | 0.006 | 0.004 | 0.12 |
| 2007 | 0.011 | 0.002 | <0.01 |
| 2008 | 0.008 | 0.002 | <0.01 |
| 2009 | 0.009 | 0.005 | 0.04 |
| 2010 | 0.012 | 0.002 | <0.01 |
| 2011 | 0.006 | 0.002 | <0.01 |
| 2012 | 0.006 | 0.001 | <0.01 |
| 2013 | 0.006 | 0.001 | <0.01 |
| 2014 | 0.007 | 0.002 | <0.01 |
|  |  |  |  |
| 1. *40- to 60-y* | | | |
| 2004 | 0.004 | 0.004 | 0.28 |
| 2005 | 0.003 | 0.003 | 0.42 |
| 2006 | -0.002 | 0.002 | 0.37 |
| 2007 | 0.002 | 0.002 | 0.24 |
| 2008 | -0.003 | 0.001 | 0.06 |
| 2009 | 0 | 0.003 | 0.89 |
| 2010 | -0.005 | 0.002 | <0.01 |
| 2011 | 0.001 | 0.001 | 0.42 |
| 2012 | -0.002 | 0.001 | 0.22 |
| 2013 | -0.002 | 0.001 | 0.17 |
| 2014 | 0 | 0.001 | 0.91 |
